# Diabetes Therapeutics Beyond Hyperglycaemia: Toward Biological Systems Redesign

**DOI:** 10.3390/biomedicines14061336

**Published:** 2026-06-12

**Authors:** Solomon Habtemariam

**Affiliations:** Pharmacognosy Research & Herbal Analysis Services UK, 124 City Road, London EC1V 2NX, UK; s.habtemariam@herbalanalysis.co.uk

**Keywords:** diabetes mellitus, systems biology, biological systems redesign, metabolic networks, precision medicine, incretin-based therapies, SGLT2 inhibitors, organ-protective pharmacology, immunometabolism, cardiovascular–renal axis, neuroendocrine regulation, closed-loop insulin systems, artificial intelligence in diabetes, network medicine, chronic disease modelling

## Abstract

Diabetes pharmacology has historically been dominated by a glucose-centric framework in which therapeutic efficacy is defined primarily by reduction in blood glucose and glycated haemoglobin (HbA1c). Although this paradigm transformed diabetes from a fatal disease into a chronic manageable condition, persistent cardiovascular, renal, inflammatory, and metabolic complications have exposed the limitations of viewing diabetes principally as a hyperglycaemic disorder. This perspective examines the progressive conceptual transition occurring across modern diabetes therapeutics, beginning with the exhaustion of the traditional glucose-centred model and extending through the emergence of incretin-based therapies, organ-protective pharmacology, immunological intervention, regenerative endocrinology, bioengineering, and AI-enabled closed-loop systems. Drawing on these developments, it argues that contemporary therapeutic advances progressively derive their efficacy from coordinated modulation of interconnected physiological networks rather than solely glucose-lowering effect. On this basis, the article proposes biological systems redesign as a unifying conceptual model for the future of diabetes therapeutics, in which treatment is directed toward the restoration of integrated metabolic and organ-level homeostasis, the preservation of system resilience, and the interception of disease progression across multiple biological scales.

## 1. Introduction

For almost a century, lowering blood glucose has remained the central focus of diabetes pharmacology. From the advent of insulin therapy to the adoption of glycated haemoglobin (HbA1c) as the primary therapeutic target, the field has largely developed within a glucose-centred paradigm in which the normalisation of glycaemia has been equated with effective disease control. This paradigm transformed diabetes from an acutely fatal disorder into a chronic manageable disease and represents as one of the most successful achievements in modern therapeutics. Despite the introduction of many potent glucose-lowering therapeutics, the global burden of cardiovascular disease, heart failure, chronic kidney disease, and obesity-associated metabolic dysfunction in diabetes has continued to rise over the last few decades [1,2]. The persistent prevalence of these complications even after improved glycaemic control highlights the fundamental limitation of the underlying conceptual framework of diabetes pharmacology.

Prospective studies and landmark clinical trials in the 1990s established that intensive glycaemic control reduces the risk of microvascular complications (though not macrovascular diseases) such as retinopathy and nephropathy [3,4,5]. Subsequent studies, however, have shown that intensive glucose lowering, despite achieving lower HbA1c levels, did not result in reduced mortality. For example, the ACCORD trial conducted in the United States found that in patients with high-risk type 2 diabetes (T2D), intensive glucose lowering to near-normal HbA1c levels over 3.5 years increased mortality without significantly reducing major cardiovascular events [6]. In other studies, only modest or delayed cardiovascular benefits were observed following intensive glycaemic control strategies. The ADVANCE trial, an international study coordinated from Australia and France involving patients with T2D, found that intensive glucose control targeting an HbA1c of 6.5% led to a modest (~10%) reduction in combined major macrovascular and microvascular events. This benefit was primarily driven by a reduction in nephropathy, with no significant effect on major cardiovascular outcomes [7]. Similarly, the clinical trial in the United States conducted in older veterans with poorly controlled T2D found that intensive glucose control did not significantly reduce major cardiovascular events, death, or most microvascular complications, except for a modest reduction in the progression of albuminuria [8]. A prospective meta-analysis of 102 studies further demonstrated that T2D is associated with approximately a two-fold-increased risk of vascular disease, independent of other risk factors. Moreover, even among patients who achieve the recommended HbA1c targets, substantial residual cardiovascular and renal risk persists [9]. Using Swedish nationwide data, Aidin Rawshani et al. [10] showed that in both type 1 diabetes (T1D) and T2D patients, improved glycaemic control is associated with a lower risk of complications. However, substantial excess mortality and cardiovascular risk persisted despite lower HbA1c levels. This diabetes burden reflects the broader pathobiology encompassing adipose tissue dysfunction, chronic low-grade inflammation, mitochondrial stress, endothelial injury, neurohormonal activation, immune dysregulation, and maladaptive inter-organ signalling [11]. On these bases, hyperglycaemia is mostly viewed not as a disease itself, but as one measurable expression of a broader, systems-level metabolic disorder. Accordingly, the reductionist assumption that hyperglycaemia alone drives the development of diabetic complications is under growing scrutiny.

**Figure 1:**
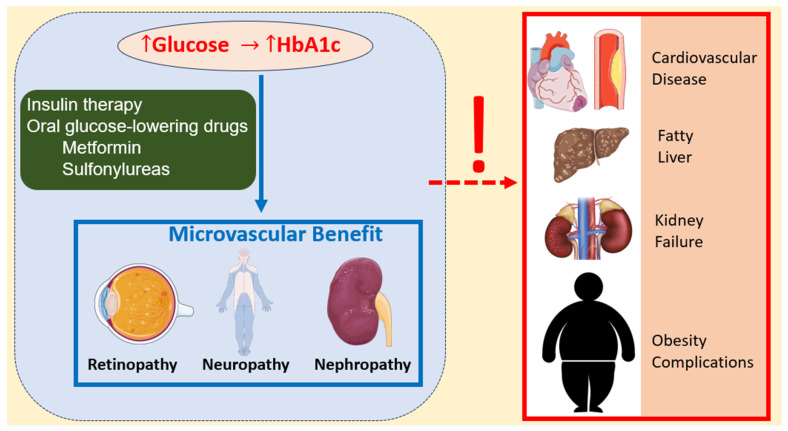
The glucose-centric paradigm of diabetes as a glycaemic disorder. The traditional glucose-centric framework of diabetes views disease severity and therapeutic success primarily defined by blood glucose reduction and HbA1c lowering. Standard glucose-lowering therapies include insulin therapy, oral glucose-lowering drugs, metformin, and sulfonylureas, all directed toward restoration of glycaemic control. Clinical benefit is predominantly conceptualised through prevention of microvascular complications, including neuropathy, retinopathy, and nephropathy, which develop over long-term disease progression. This paradigm established diabetes as a chronic endocrine disorder in which sustained glycaemic management is central to reducing downstream complication risk, particularly in relation to long-term microvascular disease burden. Up (↑) arrow denote an elevated level.

The inadequacy of the glucose-centric therapeutics approach (Figure 1) became even more evident as T2D converged biologically with obesity, fatty liver disease, heart failure, and chronic kidney disease. This convergence reframed diabetes as a multi-organ network disease rather than an isolated endocrine abnormality. A drug that lowers blood glucose levels without modifying cardiovascular trajectories, preserving renal function, improving metabolic flexibility, or attenuating inflammatory stress addresses only a partial dimension of the diabetes disease biology. Further pharmacological advances accelerated this conceptual transition. In randomised cardiovascular outcome trials, Marso et al. [12,13] showed that both liraglutide and semaglutide in patients with T2D reduced major adverse cardiovascular events, with benefits extending beyond glucose lowering, supporting cardioprotective effects of GLP-1 receptor agonists. Across these large randomised trials in T2D and heart failure populations, SGLT2 inhibitors consistently reduced heart failure hospitalisation and slowed renal disease progression, with benefits that appeared largely independent of glycaemic lowering and extended beyond glucose control to cardiorenal protection [14,15,16]. Simultaneously, immunomodulatory therapy in T1D, regenerative β-cell approaches, and artificial inelegance (AI)-enabled closed-loop systems began shifting therapeutic intent from metabolic correction toward disease intervention and biological restoration. These developments represent more than incremental drug innovation, signalling a transition toward a fundamentally different therapeutic logic. This perspective argues that diabetes pharmacology is entering a post-glycaemic era characterised by biological systems redesign as a therapeutic strategy aiming at modifying interacting metabolic, immune, endocrine, and organ-level networks rather than isolated biochemical pathways. Within this emerging paradigm, success is increasingly defined not by glucose normalisation alone, but by restoration of systemic metabolic resilience and alteration of the disease trajectory itself.

## 2. The Incretin Revolution and Metabolic Convergence

The emergence of incretin-based therapeutics represents a critical turning point in diabetes pharmacology, as they were not only effective in lowering glucose but also challenged the long-standing assumption that glucose regulation alone defines therapeutic success. GLP-1 receptor agonists demonstrated that a metabolic drug could simultaneously influence appetite regulation, body weight, cardiovascular biology, inflammation, hepatic metabolism, and renal physiology, thereby reframing diabetes treatment as multisystem metabolic modulation rather than isolated glycaemic correction.

The physiological basis of incretin was based on the observation that oral glucose elicits greater insulin secretion than intravenous glucose through gut-derived hormonal signalling [17]. Among the incretin hormones, GLP-1 emerged as a particularly compelling therapeutic target because of its integrated effects on pancreatic β-cell insulin secretion, glucagon suppression, gastric emptying, satiety signalling, and energy intake [18,19]. Drugs like GLP-1 receptor agonists were initially positioned within the traditional glucose-lowering framework, but their biological effects observed in clinical trials far exceeded expectations based solely on HbA1c reduction. Hence, liraglutide and semaglutide fundamentally altered the therapeutic landscape by demonstrating reductions in major adverse cardiovascular events independent of their glucose-lowering efficacy [12,13]. In one study, liraglutide reduced cardiovascular death, nonfatal myocardial infarction, and stroke in patients with T2D at high cardiovascular risk [12]. Similarly, semaglutide in another study produced significant reductions in cardiovascular outcomes despite relatively modest differences in glycaemic indices between treatment groups [13]. Hence, conceptually, incretin pharmacology is now seen as systemic disease biology modulation rather than merely correcting circulating glucose concentrations.

Subsequent clinical trials (e.g., REWIND) demonstrated the cardiovascular benefit with dulaglutide even in populations with lower baseline cardiovascular risk, suggesting broader disease-modifying potential beyond secondary prevention settings [20]. Semaglutide additionally produced unprecedented weight reduction approaching magnitudes previously achievable primarily through bariatric surgery [21]. The clinical implications of these findings extended far beyond diabetes management and catalysed the convergence of obesity medicine and metabolic therapeutics into a unified pharmacological domain. Obesity and diabetes share overlapping pathophysiological mechanisms including insulin resistance, adipose inflammation, altered nutrient sensing, mitochondrial dysfunction, and neuroendocrine dysregulation. They were historically, however, treated as adjacent but distinct diseases. The GLP-1 receptor agonists destabilised this separation by demonstrating that modulation of appetite regulation and energy balance could simultaneously improve glycaemia, cardiovascular outcomes, hepatic steatosis, and renal risk profiles. In this framework, obesity emerged not merely as a comorbidity of diabetes, but as a central systems-level driver of metabolic disease progression.

**Figure 2:**
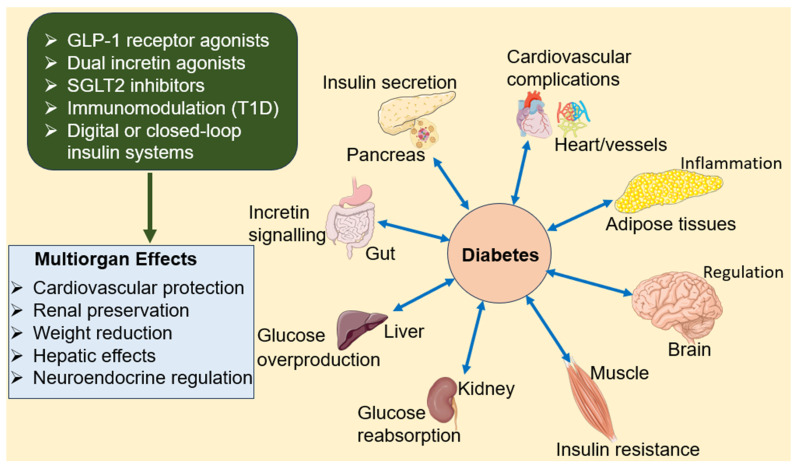
Emergence of organ-protective pharmacology beyond glycaemic endpoints. The figure depicts the transition from glucose-centred management to organ-focused therapeutic benefit. Cardiovascular, renal, metabolic, and immunological protection are represented as primary outcome domains, independent of explicit glycaemic framing. Contemporary therapeutic classes, including GLP-1 receptor agonists, SGLT2 inhibitors, immunomodulatory strategies, regenerative approaches, and closed-loop insulin delivery systems, are illustrated as agents producing multi-organ benefits across cardiovascular–renal, metabolic, immune, and neuroendocrine domains. This stage reflects a conceptual decoupling of organ protection from glycaemic control as the sole determinant of therapeutic efficacy.

The development of dual and triple incretin agonists accelerated this systems-oriented therapeutic evolution. Tirzepatide, a dual glucose-dependent insulinotropic polypeptide (GIP) and GLP-1 receptor agonist, represents the clearest prototype of multi-axis metabolic integration [22]. In SURPASS trials, tirzepatide produced profound reductions in HbA1c alongside unprecedented weight loss exceeding many existing pharmacotherapies [23,24]. The magnitude of metabolic improvement also appeared disproportionate to glucose lowering alone, implying broader reprogramming of nutrient handling, adipose biology, insulin sensitivity, and central appetite regulation. From a therapeutic point of view, the significance of incretin-based therapies lies not only in their efficacy, but in the therapeutic logic they embody. Traditional diabetes drugs targeted isolated physiological defects: insulin deficiency, hepatic gluconeogenesis, renal glucose reabsorption, and insulin resistance (Figure 1). On the other hand, incretin-based polyagonists operate across interconnected metabolic networks involving the pancreas, gastrointestinal tract, adipose tissue, liver, cardiovascular system, and central nervous system. Their success suggests that future metabolic therapeutics may derive efficacy precisely from coordinated multi-organ modulation (Figure 2) rather than selective pathway specificity. Emerging triple agonists targeting GLP-1, GIP, and glucagon receptors extend this paradigm further by attempting to engineer integrated metabolic states rather than correct single biochemical abnormalities [25]. Such approaches consistently resemble systems pharmacology, where therapeutic benefit emerges from orchestrated network-level adaptation. The goal is no longer merely the normalisation of glucose metrics, but the restoration of metabolic flexibility, energy balance, and organ resilience.

The incretin era has reshaped both regulatory expectations and scientific priorities in diabetes drug development, particularly following regulatory requirements for cardiovascular outcome trials introduced after safety concerns with earlier glucose-lowering agents such as rosiglitazone. As described for the landmark trials above [12,13], cardiovascular protection, renal preservation, weight reduction, and sustained metabolic benefits had become a primary therapeutic endpoint rather than secondary advantages. This further established a new pharmacological principle: metabolic diseases cannot be effectively managed through glucose reduction alone because they are fundamentally disorders of inter-organ communication and energy regulation. GLP-1 receptor agonists and multi-agonist therapies therefore represent more than successful drug classes as they constitute early examples of therapeutic metabolic systems engineering. Their emergence signalled the beginning of a post-glycaemic framework (Figure 1) or even organ-centric approach (Figure 2), in which diabetes treatment converges with obesity biology, cardiovascular medicine, and systems endocrinology.

## 3. Organ-Protective Pharmacology Beyond Glycaemia

Among the most disruptive developments in contemporary diabetes therapeutics has been the emergence of sodium-glucose cotransporter-2 (SGLT2) inhibitors as agents capable of modifying cardiovascular and renal outcomes independently of glycaemic control. Unlike earlier glucose-lowering drugs that were evaluated primarily through changes in HbA1c, SGLT2 inhibitors demonstrated that metabolic therapies could directly alter organ-level disease trajectories. In doing so, they fundamentally challenged the long-standing assumption that cardiovascular and renal protection in diabetes would necessarily arise as downstream consequences of improved glucose regulation.

SGLT2 inhibitors were initially developed as relatively straightforward renal glucose-lowering agents targeting proximal tubular glucose reabsorption [26]. By promoting urinary glucose excretion, these agents reduced plasma glucose concentrations through an insulin-independent mechanism and were therefore initially viewed as incremental additions to the antihyperglycaemic therapeutics. However, this interpretation changed abruptly following the EMPA-REG OUTCOME trial, in which empagliflozin was associated with an unexpected reduction in cardiovascular mortality, hospitalisation for heart failure, and all-cause mortality in patients with T2D and established cardiovascular disease [14]. Notably, the magnitude and rapid onset of cardiovascular benefit appeared disproportionate to the modest reductions in HbA1c observed during the study.

The conceptual implications of EMPA-REG trial were profound. Cardiovascular protection emerged too rapidly to be plausibly explained by delayed prevention of hyperglycaemia-related vascular injury alone, suggesting the involvement of broader haemodynamic, renal, inflammatory, and metabolic mechanisms. Subsequent trials reinforced this paradigm shift. For example, the CANVAS Programme demonstrated reductions in cardiovascular events and heart failure hospitalisation with canagliflozin [27], while the DECLARE-TIMI 58 trial confirmed significant reductions in heart failure outcomes with dapagliflozin across broader patient populations [15]. These studies revealed that SGLT2 inhibition was exerting systemic protective effects extending well beyond glycaemic regulation. Perhaps even more transformative was the emergence of robust renal protection. In the CREDENCE trial, canagliflozin significantly reduced the risk of progression to end-stage kidney disease, doubling of serum creatinine, and renal mortality in patients with diabetic nephropathy [28]. The DAPA-CKD trial subsequently demonstrated that dapagliflozin preserved renal function and reduced kidney disease progression in patients with chronic kidney disease, including those without diabetes, thereby directly decoupling nephroprotection from glucose lowering itself [29]. A similar conceptual transformation occurred in heart failure therapeutics as evidenced by the DAPA-HF and EMPEROR-Reduced trials [16,30]. The studies demonstrated that SGLT2 inhibitors reduced instances of worsening heart failure and cardiovascular death in patients with heart failure with reduced ejection fraction, regardless of the presence or absence of diabetes.

Recent systematic reviews and meta-analyses have consolidated evidence that SGLT2 inhibitors confer class-wide cardiorenal protection. In a pooled analysis of 151,023 participants, Ali et al. [31] reported reductions in cardiovascular mortality (14%), heart failure hospitalisation (30%), and composite kidney outcomes (32%), with additional benefits on all-cause mortality and major adverse cardiovascular events. Similar findings were confirmed across heart failure phenotypes and varying degrees of renal dysfunction by Hong et al. [32]. These analyses shift interpretation beyond individual trial results toward a convergent class effect operating across interconnected cardiovascular and renal pathways. Consistent with this view, Buonpane et al. [33] highlighted the fact that the benefits of SGLT2 inhibitors after myocardial infarction likely arise from coordinated effects on inflammation, endothelial function, vascular remodelling, and metabolic regulation, supporting their evolution from glucose-lowering agents to network-modifying therapies.

The proposed mechanisms of SGLT2 inhibitors include restoration of tubuloglomerular feedback, a reduction in intraglomerular pressure, natriuresis, modulation of sympathetic activity, and effects on myocardial energetics, oxidative stress, and mitochondrial function [34]. While renal haemodynamic mechanisms are supported by physiological and clinical evidence, many of the broader metabolic and cellular effects largely based on experimental and preclinical studies [35]. Nevertheless, the persistence of cardiovascular and renal benefits in non-diabetic populations suggests that no single mechanism is sufficient to explain the observed outcomes. Hence, SGLT2 inhibitors appear to induce a favourable systems-level physiological recalibration involving renal haemodynamics, cardiac function, inflammatory signalling, and metabolic substrate utilisation [33,36]. As with incretin agonists, studies on SGLT2 inhibitor support a systems-oriented view of diabetes pharmacology by challenging the notion that complications are merely downstream effects of hyperglycaemia. Instead, diabetic complications arise from interconnected maladaptive networks involving the kidney, heart, vasculature, autonomic nervous system, and energy metabolism. These agents have also reshaped clinical trial design and therapeutic evaluation, with increasing emphasis on organ protection (Figure 2) and disease modification alongside glycaemic efficacy.

## 4. Immunological Reprogramming and Disease Interception

While therapeutic innovation in T2D has progressively focused on metabolic systems integration, advances in T1D are driving an equally important conceptual transition: the movement from glycaemic replacement toward immune-mediated disease interception. Traditionally, T1D treatment has been defined by exogenous insulin replacement after irreversible β-cell destruction has already occurred. This framework positioned hyperglycaemia as the clinical starting point of the disease. T1D is now understood, however, as a progressive immune-mediated spectrum that begins years before symptomatic presentation, evolves through identifiable preclinical stages, and may be modifiable before overt metabolic failure develops [37]. This reconceptualisation emerged through longitudinal studies demonstrating that islet autoimmunity precedes clinical diabetes by prolonged periods and follows predictable trajectories associated with progressive β-cell dysfunction [38]. The presence of multiple autoantibodies confers exceptionally high lifetime risk of progression to symptomatic disease, effectively redefining T1D as an identifiable chronic autoimmune process rather than an abrupt endocrine catastrophe [39]. Consequently, therapeutic objectives began shifting from late-stage metabolic compensation toward early immunological intervention and preservation of endogenous β-cell mass.

The immunopathogenesis of T1D is now recognised as a complex systems-level interaction involving adaptive immunity, innate inflammatory signalling, genetic susceptibility, environmental triggers, and β-cell stress responses. Autoreactive CD8+ T cells play central roles in β-cell destruction, while antigen-presenting cells, cytokine networks, and regulatory T-cell dysfunction contribute to sustained autoimmune activation [40,41]. Even more crucially, β-cells themselves are now understood not as passive victims but as active participants in disease evolution through stress-induced antigen presentation and inflammatory signalling [42]. This broader systems perspective reframes T1D as a dynamic immune–endocrine network disorder rather than isolated pancreatic failure. Against this background, immunomodulatory therapies have begun to redefine therapeutic possibility. The most consequential breakthrough emerged with teplizumab, an anti-CD3 monoclonal antibody being designed to attenuate autoreactive T-cell activity and preserve β-cell function. In a landmark trial involving high-risk individuals with stage 2 T1D, teplizumab significantly delayed the progression to clinical disease, establishing for the first time that pharmacological intervention could alter the natural history of T1D before symptomatic onset [43]. Median delay to clinical diagnosis exceeded two years, representing a major conceptual advance in endocrine therapeutics.

The importance of teplizumab extends beyond its clinical efficacy. Historically, diabetes therapy began only after metabolic decompensation became clinically apparent. Teplizumab instead demonstrated that diabetes could become a target for anticipatory biological intervention. Hence, disease interception rather than disease management emerged as a viable therapeutic strategy. This shift parallels developments in oncology and cardiovascular medicine, where risk-state identification and preclinical intervention consistently define modern preventive therapeutics. Additional immunotherapeutic approaches have reinforced this trajectory. For example, trials investigating abatacept, rituximab, low-dose anti-thymocyte globulin, golimumab, and regulatory T-cell therapies have demonstrated varying degrees of β-cell preservation and immune modulation in recent-onset T1D [44,45,46,47]. Although many interventions remain partially effective or transient in benefit, they support a central principle that autoimmune diabetes is biologically modifiable. Furthermore, therapeutic responses appear to be influenced by disease stage, immune heterogeneity, age, and residual β-cell reserve, suggesting the need for precision immunology approaches rather than uniform treatment paradigms.

The integration of autoantibody profiling, genetic risk stratification, T-cell phenotyping, metabolomics, and continuous glycaemic monitoring enables the identification of individuals at distinct stages of diabetes progression [48]. This evolving capacity reframes diabetes from a reactive clinical diagnosis into a biologically multilayered disease process that may be amenable to targeted early intervention. Consequently, the therapeutic objective may shift from lifelong insulin replacement following β-cell failure toward preservation of endogenous β-cell function before irreversible tissue injury occurs. Emerging immunotherapeutic approaches further highlight the interplay between inflammatory signalling, cellular stress responses, metabolic regulation, and adaptive immunity. Within this framework, diabetes can be conceptualised through systems immunology models in which metabolic dysfunction and immune dysregulation evolve through reciprocal biological feedback mechanisms.

## 5. Regeneration, Bioengineering, and Closed-Loop Systems

The evolution of diabetes therapeutics has extended beyond pharmacology into the domains of regenerative medicine, bioengineering, and computational physiology. While traditional diabetes treatment focused primarily on correcting biochemical abnormalities through exogenous drug administration, emerging technologies aim to restore, replace, or algorithmically replicate endogenous metabolic control systems themselves. This transition represents a critical conceptual advance that diabetes management is moving from reactive metabolic correction toward engineered restoration of physiological function. Central to this transformation is the recognition that β-cell failure represents not merely a deficit in insulin availability, but the collapse of a highly dynamic glucose-sensing network integrating endocrine signalling, nutrient flux, neural regulation, and immune interactions. Exogenous insulin therapy, despite major advances in formulation and delivery, only partially reproduces the adaptive precision of endogenous β-cell physiology. Consequently, restoration of physiological glucose regulation requires reconstruction of integrated biological control systems rather than simple hormone replacement.

Efforts toward β-cell preservation and replacement have accelerated over the past decade. Islet transplantation initially demonstrated that the restoration of endogenous insulin secretion could result in near-physiological glycaemic regulation in selected individuals with T1D [49]. However, widespread application remained constrained by donor scarcity, immune rejection, and gradual graft failure. These limitations stimulated intense investigation into stem-cell-derived β-cell generation and bioengineered cellular replacement strategies. Recent advances, therefore, have focussed on pluripotent stem cell technology, which has substantially expanded the feasibility of regenerative endocrinology. Human stem cell-derived pancreatic endoderm and insulin-producing β-like cells have demonstrated functional glucose responsiveness and insulin secretion in both preclinical and early clinical studies [50,51]. Recently, stem-cell-derived islet replacement therapies have shown evidence of sustained endogenous insulin production in patients with T1D, marking an important translational milestone [52]. These approaches suggest that the restoration of intrinsic endocrine function is not merely theoretical but is becoming clinically achievable. Regenerative therapies are further evolving in parallel with advances in immune protection and tissue engineering. Encapsulation platforms designed to shield transplanted β-cells from immune destruction while preserving nutrient and oxygen exchange represent a major area of innovation [53]. Gene-editing approaches aimed at generating hypoimmunogenic stem-cell-derived islets further extend this strategy by attempting to engineer immune-evasive cellular therapeutics [54]. In this framework, diabetes treatment seems to resemble systems reconstruction, integrating cell biology, biomaterials science, immunology, and synthetic engineering into unified therapeutic architectures.

Simultaneously, technological advances in glucose sensing, insulin delivery, and AI have transformed the landscape of automated metabolic regulation. Continuous glucose monitoring (CGM) systems enabled real-time characterisation of glycaemic dynamics, exposing the limitations of intermittent glucose assessment and static therapeutic adjustment [55]. Integration of CGM with insulin pumps subsequently enabled development of hybrid and fully automated closed-loop insulin delivery systems, commonly described as artificial pancreas technologies. Landmark trials demonstrated that closed-loop systems significantly improve time in range, reduce hypoglycaemia, and enhance glycaemic stability across diverse patient populations [56,57]. Unlike conventional insulin therapy, these systems continuously adapt insulin delivery in response to fluctuating physiological states, approximating endogenous metabolic feedback regulation. Their importance lies not solely in improved glycaemic metrics, but in the emergence of programmable physiological control as a therapeutic principle.

Artificial intelligence and machine learning are further accelerating the post-glucose-centric transitions. Adaptive algorithms steadily integrate glucose variability, behavioural data, circadian patterns, nutritional inputs, and activity profiles to optimise insulin dosing in real time [58]. Future systems may incorporate multimodal biosensing, predictive metabolic modelling, and automated hormonal co-delivery involving glucagon, amylin analogues, or incretin-based therapies. As these systems evolve, diabetes management shifts from episodic intervention toward continuous computational regulation of metabolic state. The convergence of regenerative medicine and computational bioengineering carries profound conceptual implications. Instead of diabetes therapeutics seeking to compensate for failed physiology through external pharmacological support, emerging approaches aim to restore or emulate endogenous regulatory architecture itself. Stem-cell-derived β-cell systems attempt biological replacement of damaged endocrine networks, while closed-loop technologies create cybernetic analogues of physiological metabolic control.

The above-mentioned therapeutic paradigm transition also redefines the relationship between biology and technology in chronic disease management. Diabetes now serves as a prototype for integrated bio-digital therapeutics in which sensors, algorithms, engineered tissues, and adaptive drug delivery systems function as coordinated regulatory ecosystems. The therapeutic target is no longer glucose alone, but the restoration of dynamic metabolic adaptability across continuously fluctuating physiological environments. In this sense, the future of diabetes care may depend less on increasingly potent pharmacological agents and more on the engineering of adaptive biological and technological systems capable of restoring physiological regulation itself.

## 6. The Conceptual Shift: Biological Systems Redesign

**Figure 3:**
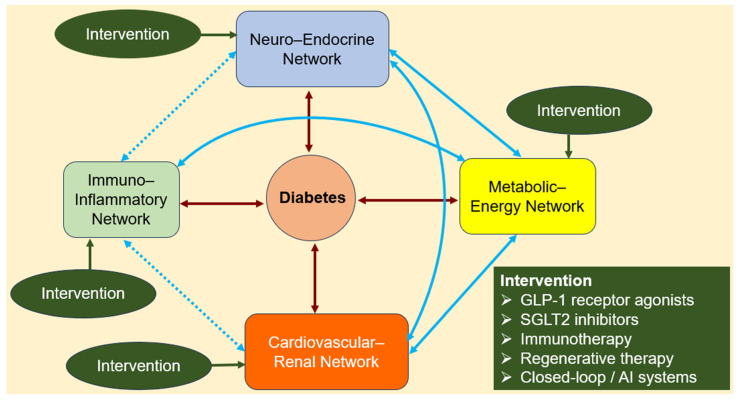
Biological systems redesign: a network-based model of diabetes pathophysiology and therapy. The figure conceptualises diabetes as a dynamic dysregulated state embedded within four interacting physiological networks: metabolic–energy homeostasis, cardiovascular–renal regulation, immune–inflammatory signalling, and neuroendocrine control. Bidirectional links between the central diabetes state and each network represent reciprocal feedback driving disease progression. Cross-network interactions reflect emergent systems-level coupling beyond organ-specific pathology. Therapeutic interventions, including incretin-based agents, SGLT2 inhibitors, immunomodulation, regenerative therapies, and closed-loop/AI systems, are represented as an external modulatory layer acting across these networks, illustrating a shift toward biological systems redesign rather than isolated glycaemic control. Solid lines indicate strong interactions, whereas dashed lines indicate comparatively weaker but yet important interactions.

As stated in the previous sections, contemporary diabetes research now views diabetes not as an isolated disorder of glycaemic regulation, but as a dynamic systems disease involving interacting metabolic, immune, endocrine, neural, cardiovascular, renal, and behavioural networks distributed across multiple organs [59,60]. This transition has become evident in modern clinical frameworks such as the American Association of Clinical Endocrinology 2023 Comprehensive T2D Management Algorithm, which prioritises obesity management, cardiovascular and renal protection, and individualised cardiometabolic risk reduction alongside glycaemic control [61]. Diabetes management is therefore progressively moving from glucose-centric (Figure 1) and an organocentric (Figure 2) paradigm toward systems-level metabolic regulation (Figure 3).

Within this emerging therapeutic model, effective therapy requires what may be described as biological systems redesign. This systems redesign refers to therapeutic modification of interacting physiological networks in order to restore adaptive metabolic homeostasis rather than merely correcting isolated biochemical abnormalities. The approach contrasts sharply with classical reductionist pharmacology, which historically pursued highly selective molecular targets under assumptions of linear disease causality. As already discussed, in diabetes, the isolated glucose-lowering approach repeatedly failed to fully eliminate cardiovascular, renal, inflammatory, and metabolic risk, exposing the limitations of single-endpoint therapeutic models [61,62]. Contemporary systems pharmacology instead proposes that durable therapeutic benefit in complex chronic disease emerges from coordinated modulation of interconnected physiological pathways rather than maximal specificity toward single molecular targets [63].

As discussed in previous sections, incretin-based therapies and SGLT2 inhibitors demonstrate that therapeutic efficacy extends well beyond glucose lowering alone. GLP-1 receptor agonists and incretin polyagonists simultaneously improve body weight, cardiovascular outcomes, inflammatory signalling, and metabolic regulation, while dual GIP/GLP-1 agonists such as tirzepatide exemplify integrated multi-pathway modulation rather than isolated receptor targeting [21,23,64]. Likewise, SGLT2 inhibitors confer cardiovascular and renal protection through broader haemodynamic, metabolic, and neurohormonal effects extending beyond glycaemic control [16,29]. These therapies are now seen to act through coordinated restructuring of cardiometabolic physiology rather than singular correction of hyperglycaemia alone. As stated above, immunotherapies such as teplizumab modify autoimmune network activity before overt disease onset, while closed-loop insulin systems integrate continuous sensing, predictive algorithms, and adaptive insulin delivery to create dynamic cybernetic metabolic regulation [43,56,57]. Regenerative and stem-cell-based approaches further extend this paradigm by seeking restoration of endogenous endocrine network functionality rather than chronic hormone replacement alone [65].

Thus, the recent advances in diabetes therapy align its therapeutics with broader principles emerging from systems biology, network medicine, and precision medicine. Systems biology conceptualises disease as dysregulation of interconnected adaptive networks characterised by feedback regulation, redundancy, and emergent behaviour rather than isolated molecular defects [66]. Network medicine similarly frames disease as perturbation of interacting biological modules spanning molecular, cellular, organ-level, and environmental systems [67]. Diabetes exemplifies this transition because its pathophysiology extends across pancreatic β-cell dysfunction, adipose inflammation, hepatic metabolism, skeletal muscle insulin resistance, gut-derived endocrine signalling, immune activation, renal haemodynamics, autonomic regulation, and central nervous system energy sensing. Hyperglycaemia therefore represents only one visible output of broader network destabilisation rather than the singular cause of disease progression.

The systems perspective shifts therapeutic goals from normalising static biomarkers (e.g., fasting glucose and HbA1c) to enhancing adaptive metabolic resilience, defined as the ability of integrated physiological systems to maintain stability under fluctuating nutritional, inflammatory, behavioural, and energetic stressors. Accordingly, therapeutic success in diabetes is now defined by cardiovascular and renal protection, improved metabolic flexibility, weight reduction, and long-term organ resilience, rather than glucose lowering alone [61,68]. A parallel conceptual shift involves movement from disease treatment toward prediction and interception. Advances in immunological staging, genomic stratification, multi-omics profiling, wearable biosensing, and continuous metabolic monitoring increasingly permit identification of preclinical disease trajectories before irreversible dysfunction develops [38]. Teplizumab demonstrated that presymptomatic intervention can delay progression to clinical T1D [43], while obesity-centred incretin therapeutics increasingly target high-risk metabolic states before advanced disease emerges [64]. With these approaches, the distinction between prevention and treatment in metabolic medicine is becoming blurred. Diabetes exhibits substantial heterogeneity in genetic susceptibility, immune phenotype, adiposity distribution, β-cell reserve, cardiovascular vulnerability, and therapeutic responsiveness. Precision medicine frameworks therefore seek stratification according to multidimensional biological profiles rather than broad diagnostic categories alone [59]. Integration of genomics, proteomics, metabolomics, microbiome analysis, wearable sensors, and machine learning may ultimately permit real-time characterisation of individual metabolic network states, enabling adaptive therapeutic modulation tailored to specific pathophysiological architectures.

Biological systems redesign therefore represents more than a theoretical framework; it is increasingly becoming an operational model for 21st-century diabetes therapeutics. Incretin polyagonists, organ-protective cardiometabolic therapies, immunological interception, regenerative endocrinology, AI-guided metabolic regulation, and digital closed-loop systems collectively signal the emergence of programmable metabolic medicine. The future trajectory of diabetes care may depend less on discovering singular breakthrough molecules and more on designing integrated biological and computational systems capable of continuously restoring physiological equilibrium across interacting metabolic networks.

## 7. Strategic Implications for Drug Discovery

**Figure 4:**
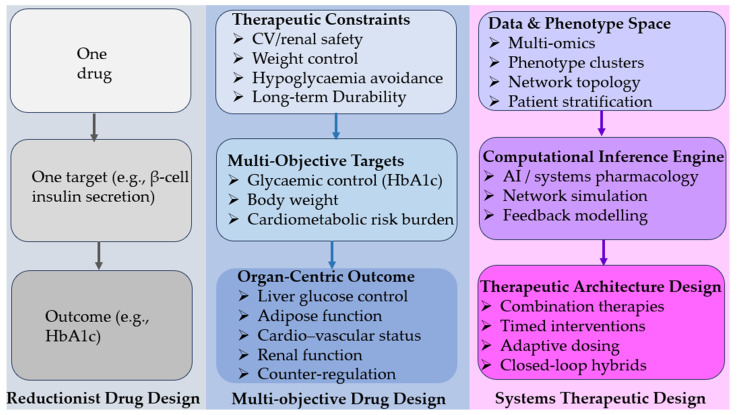
Hierarchical evolution of diabetes therapeutics from reductionist pharmacology to systems design. The hierarchical framework of diabetes therapeutics across three levels is shown. The left panel represents the reductionist paradigm, in which single drugs act on discrete molecular targets to achieve glycaemic control. The middle panel reflects an organ-centric clinical expansion, where multi-objective targets (glycaemia, weight, cardiometabolic risk) are translated into organ-linked functional outcome domains. The right panel depicts a systems therapeutic design space, integrating multi-modal data through computational and AI frameworks to generate adaptive therapeutic architectures, including dynamic combination therapies, closed-loop delivery systems, and pre-emptive interventions. The framework highlights a shift from target-based pharmacology to systems-level therapeutic engineering. CV, cardiovascular.

The evolution of diabetes therapeutics has already rendered the classical reductionist paradigm of drug discovery, defined by the principle of one target → one disease → one outcome, progressively insufficient. The transition from glucose-centric to organ-centric care has improved clinical outcomes, but it remains mechanistically incomplete. The emerging systems-design paradigm now fundamentally reframes the drug discovery objective: not to modulate isolated targets within glucose regulation, but to engineer adaptive control over distributed metabolic networks governing energy balance, inflammation, and organ resilience (Figure 4).

Incretin-based polyagonists, SGLT2 inhibitors, immunomodulators, and closed-loop metabolic systems do not derive their clinical efficacy from single-pathway specificity, but from pleiotropic network reprogramming across appetite regulation, adipose signalling, renal-cardiac coupling, immune modulation, and hepatic-muscle metabolic flux. From a drug discovery perspective, this transition demands a structural redesign of how therapeutic candidates are conceived. The dominant logic is shifting from target identification → compound optimisation → pathway suppression, toward network identification → target/mechanism orchestration → system re-stabilisation. In this framework, drug targets are no longer singular molecular entities but regulatory control points within dynamic biological networks, including metabolic switches (e.g., energy sensing pathways), inter-organ signalling axes (e.g., gut–liver–brain–kidney), and inflammation-metabolism coupling nodes (Figure 4).

Even more evident is that this systems logic expands the definition of therapeutic efficacy. As the traditional approach of endpoints such as HbA1c reduction no longer sufficient, the next-generation therapeutics must be optimised for multi-domain physiological impact, including cardioprotection, renoprotection, weight modulation, immunometabolic balance, and disease interception capacity. This aligns drug discovery with the broader cardiometabolic framework now reflected in contemporary clinical guidance such as the American Association of Clinical Endocrinology 2023 algorithm [61], where therapeutic selection is explicitly driven by multi-organ risk architecture rather than glycaemia alone.

At the methodological level, this shift is being enabled by the convergence of systems pharmacology, multi-omics integration, and computational drug design. Network medicine and systems biology now allow mapping of disease not as linear pathways but as interconnected disease modules, enabling identification of high-impact regulatory nodes whose modulation produces system-wide phenotypic reconfiguration. In parallel, AI-driven modelling, single-cell omics, metabolomics, and longitudinal digital phenotyping provide dynamic resolution of disease states, transforming drug discovery from static target selection into time-dependent system intervention design. This also reframes diabetes not as a uniform therapeutic indication but as a heterogeneous set of dynamic metabolic states. Drug discovery is therefore moving toward stratified and adaptive therapeutic engineering, where interventions are tailored not only to disease diagnosis but to the underlying network topology of metabolic dysfunction in each individual. In this context, precision medicine becomes not an adjunct but a core design principle of therapeutic innovation.

Ultimately, the strategic direction of diabetes drug discovery is shifting toward the development of integrated therapeutic systems rather than isolated pharmacological agents. Future innovation will likely emerge from combinations of pharmacological agents, biologically informed algorithms, and digitally mediated feedback systems that together function as closed-loop metabolic control architectures. In this paradigm, the drug is no longer a standalone intervention but a component within an adaptive therapeutic ecosystem designed to continuously stabilise metabolic network behaviour. Thus, we are witnessing a fundamental departure from conventional pharmacology as the objective is no longer to correct biochemical abnormalities, but to engineer controllable, resilient, and self-adapting metabolic systems capable of maintaining physiological stability across time.

## 8. Conclusions

Diabetes pharmacology is undergoing a major conceptual shift from a glucose-centred model toward systems-based therapeutic intervention. Although glycaemic control and HbA1c reduction substantially improved survival and reduced microvascular complications, persistent cardiovascular, renal, inflammatory, and metabolic risk exposed the limitations of viewing diabetes as a disorder driven solely by hyperglycaemia. The emergence of GLP-1 receptor agonists, SGLT2 inhibitors, immunomodulatory therapies, regenerative approaches, and AI-enabled closed-loop systems in recent years demonstrates that therapeutic success depends on coordinated modulation of interconnected metabolic, immune, and organ-level networks. Diabetes therapeutics are therefore entering a post-glycaemic era characterised by biological systems redesign, in which treatment aims not only to normalise biomarkers, but also to preserve organ function, enhance metabolic resilience, and prevent irreversible disease progression through integrated, adaptive therapeutic strategies.

## Data Availability

No new data were created or analysed in this study. Data sharing is not applicable to this article.
